# Leukemia in users of contemporary hormonal contraception: A nationwide registry-based cohort study among premenopausal women in Denmark

**DOI:** 10.1371/journal.pmed.1004652

**Published:** 2026-01-30

**Authors:** Caroline H. Hemmingsen, Susanne K. Kjaer, Jasmin Arvedsen, Emma O. Dahl, Amani Meaidi, Marie Hargreave, Lina S. Mørch

**Affiliations:** 1 Cancer and Medicine, Danish Cancer Institute, Copenhagen, Denmark; 2 Virus, Lifestyle and Genes, Danish Cancer Institute, Copenhagen, Denmark; 3 Department of Gynecology, Copenhagen University Hospital Rigshospitalet, Copenhagen, Denmark; Leiden University Medical Center, KINGDOM OF THE NETHERLANDS

## Abstract

**Background:**

Sex hormones have been implicated in leukemogenesis, but evidence regarding hormonal contraceptive use and leukemia risk remains limited and primarily based on older formulations. Given the widespread use of contemporary hormonal contraceptives, clarification of this potential association is needed. This study examines the association between contemporary hormonal contraceptives and leukemia risk.

**Methods and findings:**

In a nationwide cohort design, we assessed associations between the use of contemporary hormonal contraceptives and the risk of leukemia based on a cohort of all women aged 15–49 years residing in Denmark from 1995 to 2021 with no previous cancer, hysterectomy, oophorectomy, or sterilization. Information on hormonal contraception use, leukemia diagnoses, and potential confounders (age, calendar year, education) was obtained from nationwide registries. Adjusted incidence rate ratios (IRRs) and 95% confidence intervals [CIs] were estimated for any leukemia, and specific types of leukemia, associated with any hormonal contraceptive use, current and recent use, and previous use, type of product used, duration, and time since last use. Among 1,957,490 pre-menopausal women followed for 24.5 million person-years (median 12.5 years, interquartile range: 5.9,20.5), 671 were diagnosed with leukemia. The incidence rate for leukemia among current and recent users was similar to that among women who had never used hormonal contraception: IRR 0.95 (95% CI [0.78,1.16]; *p* = 0.62). No association with different durations of use was found: 0–5 years; IRR 0.93 (95% CI [0.75,1.14]; *p* = 0.48), >5–10 years; IRR 1.16 (95% CI [0.84,1.61]; *p* = 0.37), >10 years; IRR 0.67 (95% CI [0.33,1.37]; *p* = 0.27); nor for time since last use: 0–5 years; IRR 1.01 (95% CI [0.78,1.29]; *p* = 0.96), >5–10 years; IRR 1.05 (95% CI [0.76,1.45]; *p* = 0.75), >10 years; IRR 0.88 (95% CI [0.60,1.29]; *p* = 0.52). Also, the IRRs for leukemia with use of different hormonal contraceptive types (e.g., combined products; IRR 0.91 (95% CI [0.73,1.14]; *p* = 0.42) and progestin-only products; IRR 1.05 (95% CI [0.78,1.40]; *p* = 0.75)), as well as for product-specific durations of use, were for the majority close to 1. The IRRs were similar for different types of leukemia. Main study limitations include small case numbers in some analyses; therefore, additional large-scale studies are warranted to reliably exclude weak associations.

**Conclusions:**

Contemporary hormonal contraceptives were not associated with leukemia, independent of product used, duration of use, time since last use, and type of leukemia. While estimates were imprecise for some subgroups, the overall findings do not support an association.

## Author summary

**Why was this study done?:**

- Sex hormones have been implicated in the development of leukemia, but it is unclear whether hormonal contraceptive use is associated with leukemia risk.
- No study has been identified evaluating the potential impact of hormonal contraceptive use on leukemia development in women of reproductive age in the context of the contemporary formulations predominantly in use.

**What did the researchers do and find?:**

- Nearly 2 million Danish women aged 15–49 years were followed from 1995 to 2021 using Danish nationwide registry data. Women with prior cancer, hysterectomy, oophorectomy, or sterilization were excluded.
- No association was found between contemporary hormonal contraceptive use and the risk of leukemia. Results were consistent across contraceptive types, durations of use, time since last use, and leukemia types.

**What do these findings mean?:**

- These findings suggest that contemporary hormonal contraceptive use is not associated with the risk of leukemia in women of reproductive age, offering reassurance about the safety of modern hormonal contraceptives commonly used with respect to this specific cancer risk. However, weak associations cannot be ruled out, as our nationwide study lacks statistical precision to detect weak associations, given the relative rarity of leukemia.

## Abstract 3

Using Danish nationwide registry data of nearly 2 million women, Caroline H. Hemmingsen and colleagues evaluate the risk of leukemia among pre-menopausal women using contemporary hormonal contraceptives.

## Introduction

Leukemia accounts for ~2.5% of all primary cancers globally, with a 5-year survival rate in women varying from 96% to 32% depending on the subtype [1,2]. Only a few established risk factors are known, accounting for a small fraction of adult cases [3,4]. Sex hormones have been suggested to play a role in leukemogenesis [5–8]. Estrogen promotes hematopoietic stem cell self-renewal via estrogen receptor α, with pronounced effects in females and during pregnancy, as shown in mice [9]. In humans, estrogen also regulates hematopoietic cell proliferation and differentiation, and binding sites for both estrogen and progesterone receptors have been identified in leukemic cells [5,6,9]. Hormonal contraceptives suppress the hypothalamic–pituitary–gonadal axis, reducing circulating follicle-stimulating hormone (FSH) and luteinizing hormone (LH). Receptors for these gonadotropins are expressed on human hematopoietic and leukemic cells, where FSH and LH have been shown to stimulate malignant cell proliferation and migration [7]. Finally, maternal hormonal contraception use has been linked with childhood leukemia risk, supporting a potential role for sex hormones in leukemogenesis [8].

Globally, more than 400 million women aged 15–49 years use hormonal contraception [10], including approximately 40% of all Danish women in this age group [11]. Despite its widespread use and biological plausibility for a link with leukemia, only very few studies, with varying designs, have explored this association [12–15]. Notably, most of the earlier studies primarily focused on older formulations of hormonal contraceptives with higher estrogen doses and different progestins than those commonly prescribed today. Consequently, these studies largely assessed the effects of long-past exposures to older formulations, which limits the applicability of their findings to current contraceptive use. Moreover, these studies were based on few exposed cases, self-reported information, some with a case-control study design prone to selection bias, and the inclusion of women aged >50 years, potentially influenced by postmenopausal hormonal treatment.

To address this knowledge gap, the present nationwide cohort study evaluates the risk of leukemia specifically among pre-menopausal women using contemporary hormonal contraceptives.

## Materials and methods

### Study population

A nationwide registry-based cohort study was established, including all women living in Denmark aged 15–49 years on January 1, 1995, and those who subsequently were 15 years of age before December 31, 2021. In Denmark, each resident is assigned a unique personal identification number in the Civil Registration System [16], which allowed linkage of individual-level information across the following Danish nationwide registries: (1) The National Patient Registry, including all hospital diagnoses since 1978 [17]; (2) the National Prescription Registry, providing data on all pharmacy-dispensed prescriptions since 1995 [18]; (3) the Cancer Registry, containing information on all incident cancer diagnoses since 1943 [19]; (4) the Medical Birth Registry, including information on all births since 1973 [20]; and (5) Statistics Denmark, storing information about education (S1 Table).

The prescription registry is complete from 1995 onward, which marks the start year of the study. Women were excluded if they, before entry (January 1st, 1995, or their 15th birthday in 1995–2021), had been diagnosed with cancer (except nonmelanoma skin cancer) or undergone hysterectomy, oophorectomy, or sterilization. To ensure sufficient information in the registries, residency in Denmark for five years before study entry was required.

### Hormonal contraception

We assessed hormonal contraceptive use by extracting information on redeemed prescriptions in the Danish National Prescription Registry using the Anatomical Therapeutic Chemical (ATC) codes G03A*, G02BA03, G02BB01, and G03HB01 (S2 Table). The nationwide register contains complete individual-level data on all redeemed prescriptions for all Danish citizens. Each prescription record includes the type of product, date of redemption, dosage, size, and number of packages dispensed. Hormonal contraceptive use was modeled as a time-varying variable based on daily updated information on redeemed prescriptions during follow-up. The date of a filled prescription was defined as the first day of use, and the duration of use was based on the size and number of packages dispensed. To account for delayed initiation and for users taking fewer pills per day than prescribed, all durations of filled prescriptions were extended by 28 days. Hormonal contraceptive status was altered at the date when a product was initiated, discontinued, or the type of product changed, and was categorized as follows: *Current and recent use*, starting from date of filled prescription until six months after the expected end of supply, with the six-month extension chosen to account for potential diagnostic delay, i.e., the period preceding a leukemia diagnosis during which nonspecific early symptoms may have occurred, prompting medical consultation and diagnostic evaluation, all prior to registration of the diagnosis in the health record; *previous use*, started more than six months after the calculated end of supply, i.e., the time after current and recent use; *ever use*, comprising both current and recent plus previous use; and *never use*, referring to the period before any prescription for hormonal contraception had been redeemed. Exposure was further stratified by hormonal content (combined estrogen–progestin, and progestin-only), route of administration (oral, and non-oral), duration of use (0–5 years, >5–10 years, and >10 years), and time since last use (0–5 years, >5–10 years, and >10 years).

### Leukemia

In the Danish Cancer Registry, we identified the date of any incident leukemia using the International Classification of Diseases, 10th revision (ICD-10) codes classified by the Association of the Nordic Cancer Registries (NORDCAN) as “any leukemia” (ICD-10: C91-C93 C94.0, C94.2-C94.4, C94.7, C95), “acute lymphatic leukemia” abbreviated “ALL” (C91.0), “acute myeloid leukemia” abbreviated “AML” (ICD-10: C92.0, C92.3-C92.6, C93.0, C94.0, C94.2), “chronic lymphatic leukemia” abbreviated “CLL” (ICD-10: C91.1), and “chronic myeloid leukemia” abbreviated “CML” (ICD-10: C92.1, C93.1) [19,21]. Information on leukemia was updated daily throughout the follow-up, allowing for precise temporal alignment between changes in exposure status and the occurrence of leukemia.

### Other covariates

From Statistics Denmark, we obtained information on the educational level. The National Patient Registry provided hospital discharge diagnoses of polycystic ovary syndrome, endometriosis, and obesity. We also included information on infertility diagnoses from the National Patient Registry, supplemented with information on the use of fertility drugs from the National Prescription Registry. Finally, we retrieved information on smoking and body mass index collected in the first trimester of pregnancy and parity from the Medical Birth Registry and the National Patient Registry (S1 Table).

### Statistical analyses

Each woman aged 15–49 years residing in Denmark was followed from 1st of January 1995 or when she turned 15 after this date and until leukemia diagnosis, diagnosis of other cancers (except non-melanoma skin cancer), time of hysterectomy, oophorectomy, sterilization, emigration, death, 50th birthday, or end of follow-up in the registers, i.e., December 31, 2021, whichever occurred first. Follow-up was limited to December 31, 2021, corresponding to the most recent validated data available in the Danish Cancer Register. A woman was temporarily censored during pregnancy and for six months after childbirth. Poison regression models were applied to calculate incidence rate ratios (IRRs) and 95% confidence intervals [95% CI] for any leukemia and types of leukemia (ALL, AML, CLL, and CML). We estimated the incidence rate of leukemia associated with ever use, current and recent use, and previous use, compared with the incidence rate associated with never use. The IRR of leukemia was further evaluated according to the type of hormonal contraceptive used, duration, and time since last use.

Five-year age bands were used as a time scale in the Poisson regression. All main analyses included time-dependent variables for exposure to hormonal contraception, age, calendar year, and education.

To test the robustness of the results, explorative sensitivity analyses were conducted with models also adjusted for the following time-varying variables: Polycystic ovary syndrome, endometriosis, obesity, infertility, and parity. These are factors that are known to influence hormonal exposure patterns. Also, sensitivity analyses among parous women exclusively (*N* = 1,166,999) were conducted, with additional adjustment for pre-pregnancy body mass index and smoking (available for 51% and 40% women) to assess if these factors affected the results among parous women. In the Danish health registries, information on body mass index and smoking is only collected during the first trimester of pregnancy. Another sensitivity analysis was performed, restricted to the study entry years 2000–2021, to test whether longer exposure histories in the prescription registry before study entry affected the results. The start year of 2000 was chosen to ensure a minimum of five years of exposure history, given that the prescription registry has been complete since 1995. In a post hoc analysis, we assessed the association between the duration of oral contraceptive use and the risk of AML for comparability with a previous study [13]. Also, post hoc, the association between age and incident leukemia was assessed to test if the study was able to detect known associations with leukemia risk*.* Finally, post hoc, all main analyses were conducted controlling only for age (as the underlying time scale), to enable comparison with adjusted estimates. No information was missing for the main models. All analyses were conducted using R version 4.3.2. Small cell suppression was applied in accordance with data protection guidelines from Statistics Denmark to prevent identification of individuals. Specifically, event counts below five are not permitted to be displayed and must not be deducible through aggregation across table categories. Therefore, some larger numbers (i.e., greater than five) were also rounded to ensure compliance. This study follows the STROBE reporting guideline (S14 Table—STROBE checklist).

## Ethics statement

According to Danish law, studies utilizing national registry data do not require ethical approval or patient consent.

## Results

**Fig 1:**
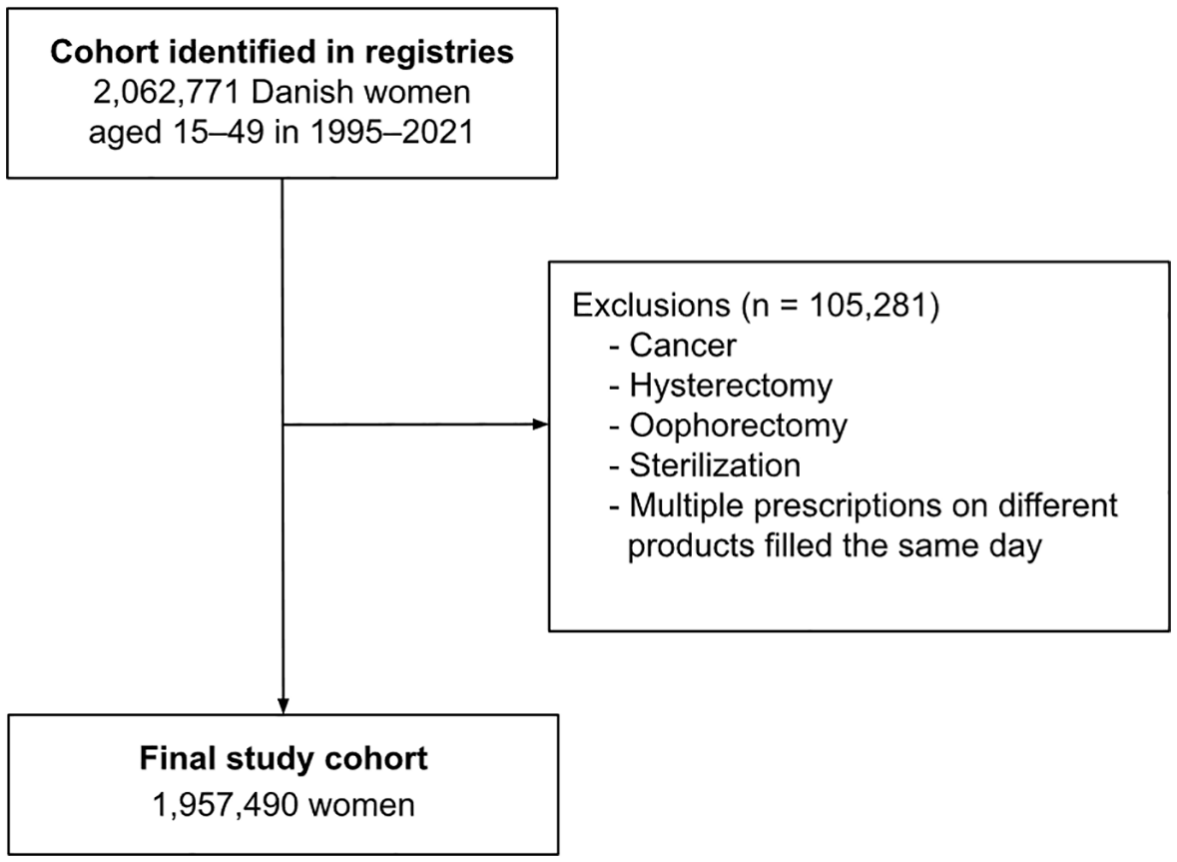
Flowchart.

A total of 1,957,490 women aged 15–49 years were included in the study population after the exclusion of women who previously had been diagnosed with cancer (except nonmelanoma skin cancer) or undergone hysterectomy, oophorectomy, or sterilization (Fig 1). During 24,495,350 person-years of follow-up (median 12.5 years [interquartile range: 5.9–20.5]), 671 women were diagnosed with incident leukemia. Of these diagnoses, 124 were ALL, 266 were AML, 100 were CLL, and 119 were CML. Overall, women with current and recent use of hormonal contraception were generally younger, more often nulliparous, and had a longer education than women who had never used hormonal contraceptives (Table 1).

**Table 1:** Characteristics of study population: 1,957,504 women aged 15–49 years during 1995–2021 in Denmark.

Characteristics	Hormonal contraceptive use						
	Never use	Current and recent use					Previous use
		Any	Combined oral	Combined non-oral	Progestin-only oral	Progestin-only non-oral	
**Person-years** per 100,000	78.6	106.9	83.5	1.4	5.1	16.8	59.4
**Age** *median (IQR)*	35 (18–43)	26 (20–35)	24 (19–31)	25 (21–31)	31 (23–39)	38 (31–43)	35 (29–42)
**University education** *%*	24.6	31.7	29.1	40.4	39.1	41.7	37.4
**Nulliparous** %	49.3	60.2	69.4	65.7	43.4	19.2	30.4
**PCOS** %	0.2	0.6	0.5	0.7	0.8	1.0	1.2
**Endometriosis** %	0.5	0.8	0.6	0.8	1.7	1.4	1.4
**Obesity** %	1.4	3.1	1.9	3.5	5.8	8.2	5.9
**Infertility** %	6.3	3.4	2.2	3.0	5.4	9.1	11.7

PCOS (ICD-8: 25690*; ICD-10: E282*), endometriosis (ICD-8: 6253*; ICD-10: N80*), obesity (ICD-8: 27799*, 27809*; ICD-10: E66* excluding E660A), infertility (ICD-8: 628*; ICD-10: N97*; ATC: G03G*).

Abbreviations: ATC, Anatomical Therapeutic Chemical classification system; ICD-8, International Classification of Diseases, 8th revision; ICD-10, International Classification of Diseases, 10th revision; IQR, Interquartile range; PCOS, Polycystic ovary syndrome.

### Any hormonal contraception

Ever use of any hormonal contraception was not associated with the risk of any leukemia, compared with never use (IRR 0.97, 95% CI [0.81,1.16]; *p* = 0.72). Neither current and recent use of any hormonal contraception, nor previous use, showed any association with leukemia risk (IRR 0.95, 95% CI [0.78,1.16]; *p* = 0.62: IRR 0.99, 95% CI [0.80,1.23]; *p* = 0.94) (Table 2). Estimates for current and recent use of any hormonal contraception were similar for specific leukemia types: ALL (IRR 1.01, 95% CI [0.65,1.58]; *p* = 0.96), AML (IRR 0.99, 95% CI [0.71,1.36]; *p* = 0.93), CLL (IRR 0.87, 95% CI [0.51,1.47]; *p* = 0.6), or CML (IRR 0.99, 95% CI [0.61,1.58]; *p* = 0.96) (Table 3).

**Table 2:** IRRs [95% CIs] for leukemia in Danish women aged 15–49 years, according to hormonal contraceptive use.

	Any leukemia		
**Hormonal contraceptive use**	**PY/100,000**	**Cases**	**IRR [95% CI]** ^*^
**Never use**	78.6	241	1 [reference]
**Ever use**	166.3	430	0.97 [0.81,1.16]
**Current and recent use**	106.9	243	0.95 [0.78,1.16]
*Combined products*	84.9	<175	0.91 [0.73,1.14]
Oral	83.5	<170	0.91 [0.73,1.14]
Non-oral	1.4	<5	0.94 [0.30,2.95]
*Progestin-only products*	22.0	<75	1.05 [0.78,1.40]
Oral	5.1	<15	0.95 [0.54,1.68]
Non-oral	16.8	60	1.07 [0.79,1.46]
**Previous use**	59.4	187	0.99 [0.80,1.23]
*Combined products*	52.6	163	1.00 [0.80,1.25]
*Progestin-only products*	6.8	24	1.02 [0.66,1.57]

Recent use: The six months following cessation of hormonal contraceptive use, as recorded in the prescription register.

Small cell suppression was applied in accordance with data protection guidelines from Statistics Denmark to prevent identification of individuals.

*Adjusted for calendar year, age, and education.

Abbreviations: CI, Confidence interval; IRR, Incidence rate ratio; PY, Person-years.

**Table 3:** IRRs [95% CIs] for types of leukemia in Danish women aged 15–49 years, according to hormonal contraceptive use.

		ALL		AML		CLL		CML	
**Hormonal contraceptive use**	**PY/100,000**	**Cases**	**IRR [95% CI]** ^*^	**Cases**	**IRR [95% CI]** ^*^	**Cases**	**IRR [95% CI]** ^*^	**Cases**	**IRR [95% CI]** ^*^
**Never use**	78.6	39	1 [reference]	91	1 [reference]	47	1 [reference]	42	1 [reference]
**Ever use**	166.3	85	1.06 [0.70,1.62]	175	1.10 [0.82,1.47]	53	0.71 [0.45,1.14]	77	0.88 [0.57,1.36]
**Current and recent use**	106.9	56	1.01 [0.65,1.58]	93	0.99 [0.71,1.36]	28	0.87 [0.51,1.47]	47	0.99 [0.61,1.58]
*Combined products*	84.9	44	0.96 [0.60,1.53]	<75	1.02 [0.72,1.44]	10	0.69 [0.34,1.40]	<35	0.99 [0.59,1.66]
Oral	83.5	44	0.97 [0.61,1.54]	<75	1.02 [0.72,1.44]	10	0.70 [0.35,1.42]	<35	0.96 [0.57,1.61]
Non-oral	1.4	0	.	<5	0.88 [0.12,6.42]	0	.	<5	3.42 [0.79,14.75]
*Progestin-only products*	22.0	<15	1.30 [0.64,2.64]	<25	0.88 [0.52,1.49]	<20	1.06 [0.56,2.00]	<20	0.98 [0.51,1.91]
Oral	5.1	<5	1.25 [0.38,4.16]	<5	0.83 [0.30,2.30]	<5	0.43 [0.06,3.15]	<5	1.44 [0.50,4.16]
Non-oral	16.8	<10	1.32 [0.60,2.89]	<20	0.89 [0.50,1.57]	<20	1.17 [0.61,2.24]	<15	0.89 [0.43,1.84]
**Previous use**	59.4	<30	1.20 [0.70,2.06]	82	1.27 [0.91,1.78]	<30	0.58 [0.34,1.02]	30	0.75 [0.44,1.27]
*Combined products*	52.6	<30	1.29 [0.75,2.23]	71	1.24 [0.87,1.75]	<25	0.60 [0.34,1.08]	25	0.72 [0.41,1.24]
*Progestin-only products*	6.8	<5	0.75 [0.18,3.23]	11	1.45 [0.75,2.80]	<5	0.60 [0.21,1.77]	5	0.98 [0.37,2.59]

Recent use: The six months following cessation of hormonal contraceptive use, as recorded in the prescription register.

Small cell suppression was applied in accordance with data protection guidelines from Statistics Denmark to prevent identification of individuals.

*Adjusted for calendar year, age, and education.

Abbreviations: ALL, Acute lymphatic leukemia; AML, Acute myeloid leukemia; CLL, Chronic lymphatic leukemia; CML, Chronic myeloid leukemia; CI, Confidence interval; IRR, Incidence rate ratio; PY, Person-years.

### Type of hormonal contraception

Current and recent use of combined estrogen-progestin was not associated with the risk of any leukemia (IRR 0.91, 95% CI [0.73,1.14]; *p* = 0.42) when compared with never use (Table 2), and neither was use of progestin-only contraceptives (IRR 1.05, 95% CI [0.78,1.40]; *p* = 0.75). Route of administration (oral versus non-oral) of both combined and progestin-only contraceptives showed similar estimates (Table 2). Estimates did not vary notably by specific types of leukemia (Table 3).

### Duration of use and time since last use

Different durations of any hormonal contraceptive use were not associated with IRRs of leukemia (Table 4). Similarly, no associations were seen between the duration of combined or progestin-only contraceptive use and the risk of leukemia (Table 4). Time since last use of any hormonal contraception was also not associated with the risk of any leukemia (Table 4).

**Table 4:** Use of hormonal contraception by duration of use and time since last use and the risk of leukemia.

	Any leukemia		
Hormonal contraceptive use	PY/100,000	Cases	IRR [95% CI]^*^
**Never use**	78.6	241	1 [reference]
**Duration of use**			
**Any hormonal contraception**			
0–5 years	88.0	185	0.93 [0.75,1.14]
>5–10 years	15.6	50	1.16 [0.84,1.61]
>10 years	3.2	8	0.67 [0.33,1.37]
***Combined***			
0–5 years	69.7	125	0.87 [0.68,1.10]
>5–10 years	12.5	<40	1.27 [0.89,1.82]
>10 years	2.7	<10	0.64 [0.28,1.45]
***Progestin-only***			
0–5 years	18.3	60	1.08 [0.80,1.48]
>5–10 years	3.1	<15	0.92 [0.49,1.71]
>10 years	0.5	<5	0.85 [0.21,3.46]
**Time since last use**			
**Any hormonal contraception**			
0–5 years	36.6	102	1.01 [0.78,1.29]
>5–10 years	14.0	51	1.05 [0.76,1.45]
>10 years	8.9	34	0.88 [0.60,1.29]

Small cell suppression was applied in accordance with data protection guidelines from Statistics Denmark to prevent identification of individuals.

*Adjusted for calendar year, age, and education.

Abbreviations: CI, Confidence interval; IRR, Incidence rate ratio; PY, Person-years.

### Additional analyses

Findings remained consistent across unadjusted and adjusted analyses, after further adjusting for other potential confounders, including polycystic ovary syndrome, endometriosis, infertility, obesity, parity, body mass index, and smoking, and when limiting the study entry to the years 2000–2021 (S3–S8 Tables and S11–S13 Tables). For comparison with a previous study, duration analysis of oral contraceptive use and AML risk was performed, showing no association (S9 Table). Finally, the known association between age and incidence of leukemia was confirmed, supporting the internal validity of the study (S10 Table).

Of note, this nationwide cohort had limited statistical precision to detect weak associations, given the relative rarity of leukemia.

## Discussion

This large cohort study, including nearly 2 million women of reproductive age, showed a reassuring finding of no increased leukemia risk with current and recent or previous use of any type of contemporary hormonal contraception. The results remained consistent across different types of leukemia, types of contraceptives, durations of use, and time since last use. For less commonly used products, the estimates were imprecise and warrant further investigation.

Only very few studies have previously assessed the association between hormonal contraceptive use and leukemia risk, with conflicting results [12–15]. Discrepancies among studies may reflect differences in methodological approaches, population characteristics, and the fact that earlier studies primarily examined older contraceptive formulations. Moreover, previous studies often included older populations, thereby primarily capturing long-past exposure, which is unlikely to reflect risks associated with more recent or current use. Consistent with our findings is one cohort study of Finnish pre-menopausal women aged 30–49 years using intrauterine devices for menorrhagia treatment, which found no association with any leukemia (standardized incidence ratio 0.93, 95% CI [0.64,1.29]) [15]. Two studies, a case-control study including both pre- and postmenopausal women and a cohort study restricted to postmenopausal women, specifically examined the risk of AML and found no increased risk with any oral contraceptive use [13,14]. However, the case-control study, suggested a decreased leukemia risk with more than five years of oral contraceptive use (odds ratio 0.55, 95% CI [0.32,0.96]) [13]. Both studies [13,14], however, relied on self-reported exposure data, which can be subject to differential [13] or non-differential [14] misclassification of exposure. The current study among reproductive-aged women (15–49 years) showed no association between the duration of oral contraceptive use and AML risk (S9 Table). One case-control study [12] reported an increased odds ratio for any acute leukemia associated with any hormonal contraceptive use more than one year before diagnosis (1.8, 95% CI [0.8,4.0]). However, the CI was notably wide and included 1, rendering the result uncertain. No association with a longer duration of use was found. This result was based on eight exposed cases and a study population with a median age of 55 years. Moreover, the products used were older oral contraceptives containing higher estrogen doses, which limit generalizability to effects among premenopausal women using contemporary types of hormonal contraceptives.

This study is, to our knowledge, the largest to date investigating the association between hormonal contraception use and leukemia risk. Distinct from most previous studies, we assessed the association specifically among women during the reproductive years. The exposure and outcome data are considered highly complete and valid because all dispensed hormonal contraceptive prescriptions are automatically and electronically recorded in the prescription registry [18] and reporting to the cancer registry is mandatory by law [19].

However, women who used hormonal contraception only before the start of the prescription registry (1995) may have been incorrectly categorized as never-users. This misclassification could potentially have led to an underestimation of the risk estimates. However, when restricting the study entry years to 2000–2021—thereby ensuring more complete exposure information—the results remained virtually unchanged. Hence, this non-differential misclassification is not expected to have any notable impact on the overall results.

Residual confounding is possible. Nevertheless, our findings remained consistent after adjusting for potential differences in characteristics of the women using versus not using hormonal contraception, including age, calendar year, education, obesity, parity, polycystic ovary syndrome, endometriosis, infertility, body mass index, and smoking. Few other risk factors for leukemia are known, including benzene exposure, ionizing radiation, genetic syndromes, and a family history of leukemia in first-degree relatives [1]. These factors are all rare in the population and are not known to be associated with hormonal contraceptive use. Furthermore, approximately 40% of women of reproductive age in Denmark use hormonal contraception [11]; thus, it is unlikely that these relatively rare risk factors are distributed differently between users and non-users to an extent where it would be possible to substantially confound the results.

Establishing that an association is precisely null (i.e., a risk estimate of exactly 1.00) requires exceptionally high statistical precision and is rarely feasible in practice. Although most point estimates were close to 1.00, the possibility of a true, albeit weak, association cannot be excluded, as it may still lie within the CI. Nonetheless, for the most commonly used products (oral combined estrogen–progestin products), the upper bound of the CI did not exceed 1.14, suggesting that any potential risk is likely to be weak.

The results of this study reassuringly suggest that there is no increased risk of leukemia with the use of contemporary types of hormonal contraceptives, disregarding duration of use and time since last use. While estimates were imprecise for less commonly used products, the overall findings for the most commonly used hormonal contraceptives do not support an association.

## Supporting information

S1 Table Overview of variables from Danish nationwide population-based registries used in the study. * Height and weight information available from 2003. ** Smoking information available from 1991.*** Education information available from 1981. Abbreviations: ICD, International Classification of Diseases.
(DOCX)

S2 Table Exposure classification and ATC codes. Abbreviations: ATC, Anatomical Therapeutic Chemical classification system; IUD, Intrauterine device.
(DOCX)

S3 Table IRRs [95% CIs] for any leukemia in Danish women aged 15–49 years, according to hormonal contraceptive use and adjusted for other potential confounders. *Adjusted for calendar year, age, education, parity, polycystic ovarian syndrome, endometriosis, infertility, and obesity. Abbreviations: CI, Confidence interval; IRR, Incidence rate ratio; PY, Person-years. Recent use: The six months following cessation of hormonal contraceptive use, as recorded in the prescription register. Small cell suppression was applied in accordance with data protection guidelines from Statistics Denmark to prevent identification of individuals.
(DOCX)

S4 Table IRRs [95% CIs] for types of leukemia in Danish women aged 15–49 years, according to hormonal contraceptive use and adjusted for other potential confounders. *Adjusted for calendar year, age, education, parity, polycystic ovarian syndrome, endometriosis, infertility, and obesity. Abbreviations: ALL, Acute lymphatic leukemia; AML, Acute myeloid leukemia; CLL, Chronic lymphatic leukemia; CML, Chronic myeloid leukemia; CI, Confidence interval; IRR, Incidence rate ratio; PY, Person-years. Recent use: The six months following cessation of hormonal contraceptive use, as recorded in the prescription register. Small cell suppression was applied in accordance with data protection guidelines from Statistics Denmark to prevent identification of individuals.
(DOCX)

S5 Table IRRs [95% CIs] for leukemia in Danish parous women aged 15–49 years, according to hormonal contraceptive use, adjusted for body mass index. *Adjusted for calendar year, age, and education. **Adjusted for calendar year, age, education, and body mass index in the first trimester of pregnancy. Abbreviations: CI, Confidence interval; IRR, Incidence rate ratio; PY, Person-years. Recent use: The six months following cessation of hormonal contraceptive use, as recorded in the prescription register.
(DOCX)

S6 Table IRRs [95% CIs] for leukemia in Danish parous women aged 15–49 years, according to hormonal contraceptive use, adjusted for smoking. *Adjusted for calendar year, age, and education. **Adjusted for calendar year, age, education, and smoking in the first trimester of pregnancy. Abbreviations: CI, Confidence interval; IRR, Incidence rate ratio; PY, Person-years. Recent use: The six months following cessation of hormonal contraceptive use, as recorded in the prescription register.
(DOCX)

S7 Table IRRs [95% CIs] for any leukemia in Danish women aged 15–49 years in 2000–2021, according to hormonal contraceptive use. *Adjusted for calendar year, age, and education. Abbreviations: CI, Confidence interval; IRR, Incidence rate ratio; PY, Person-years. Recent use: The six months following cessation of hormonal contraceptive use, as recorded in the prescription register. Small cell suppression was applied in accordance with data protection guidelines from Statistics Denmark to prevent identification of individuals.
(DOCX)

S8 Table IRRs [95% CIs] for types of leukemia in Danish women aged 15–49 years in 2000–2021, according to hormonal contraceptive use. *Adjusted for calendar year, age, and education. Abbreviations: ALL, Acute lymphatic leukemia; AML, Acute myeloid leukemia; CLL, Chronic lymphatic leukemia; CML, Chronic myeloid leukemia; CI, Confidence interval; IRR, Incidence rate ratio; PY, Person-years. Recent use: The six months following cessation of hormonal contraceptive use, as recorded in the prescription register. Small cell suppression was applied in accordance with data protection guidelines from Statistics Denmark to prevent identification of individuals.
(DOCX)

S9 Table Duration of recent and current use of oral contraceptives and the risk of acute myeloid leukemia. *Adjusted for calendar year, age, and education. Abbreviations: CI, Confidence interval; IRR, Incidence rate ratio; PY, Person-years. Small cell suppression was applied in accordance with data protection guidelines from Statistics Denmark to prevent identification of individuals.
(DOCX)

S10 Table IRRs [95% CIs] for leukemia in Danish women according to age group. *Adjusted for calendar year and education. Abbreviations: CI, Confidence interval; IRR, Incidence rate ratio; PY, Person-years.
(DOCX)

S11 Table IRRs [95% CIs] for leukemia in Danish women aged 15–49 years, according to hormonal contraceptive use and unadjusted. * Using age as the underlying time scale in the Poisson regression. Abbreviations: CI, Confidence interval; IRR, Incidence rate ratio; PY, Person-years. Recent use: The six months following cessation of hormonal contraceptive use, as recorded in the prescription register. Small cell suppression was applied in accordance with data protection guidelines from Statistics Denmark to prevent identification of individuals.
(DOCX)

S12 Table IRRs [95% CIs] for types of leukemia in Danish women aged 15–49 years, according to hormonal contraceptive use and unadjusted. * Using age as the underlying time scale in the Poisson regression. Abbreviations: ALL, Acute lymphatic leukemia; AML, Acute myeloid leukemia; CLL, Chronic lymphatic leukemia; CML, Chronic myeloid leukemia; CI, Confidence interval; IRR, Incidence rate ratio; PY, Person-years. Recent use: The six months following cessation of hormonal contraceptive use, as recorded in the prescription register. Small cell suppression was applied in accordance with data protection guidelines from Statistics Denmark to prevent identification of individuals.
(DOCX)

S13 Table Use of hormonal contraception by duration of use and time since last use and the risk of leukemia and unadjusted. * Using age as the underlying time scale in the Poisson regression. Abbreviations: CI, Confidence interval; IRR, Incidence rate ratio; PY, Person-years. Small cell suppression was applied in accordance with data protection guidelines from Statistics Denmark to prevent identification of individuals.
(DOCX)

S14 Table STROBE Statement—Checklist of items that should be included in reports of cohort studies, available at https://www.strobe-statement.org/, licenced under CC BY 4.0. (DOCX)

## Abbreviations

ATC: Anatomical Therapeutic Chemical
CI: confidence interval
FSH: follicle-stimulating hormone
ICD-10: International Classification of Diseases, 10th revision
IRRs: incidence rate ratios
LH: luteinizing hormone
