# Supplementary material for: Leukemia in users of contemporary hormonal contraception: A nationwide registry-based cohort study among premenopausal women in Denmark
Source: PLoS Med. 2026 Jan 30;23(1):e1004652. doi: 10.1371/journal.pmed.1004652 (PMC12875577; doi:10.1371/journal.pmed.1004652)
Supplement: S1 Table — * Height and weight information available from 2003. ** Smoking information available from 1991.*** Education information available from 1981. Abbreviations: ICD, International Classification of Diseases. (DOCX) [file pmed.1004652.s001.docx]

| S1 Table. Overview of variables from Danish nationwide population-based registries used in the study. | | | | |
| --- | --- | --- | --- | --- |
| Registry | **Established** | **Complete** | **Information included in study** | **Description** |
| Danish Civil Registration System | 1968 | 1969 | Birthday, date of death, migration | Provides comprehensive and accurate information on all individuals alive and residing in Denmark, including name, gender, date of birth, citizenship, and vital status.(1) |
| Danish Cancer Registry | 1942 | 1943 | Cancer diagnoses | Contains information on all cancer diagnoses and is considered highly valid and complete. Reporting to the Cancer Registry is mandatory.(2) |
| National Patient Registry | 1976 | 1977 | Infertility, polycystic ovary  syndrome,  endometriosis, obesity, hysterectomy, oophorectomy, sterilization | Includes information on all inpatient admissions at Danish hospitals since 1977 and outpatient visits since 1995. Diagnoses were recorded using the International Classification of Diseases, 8th Revision (ICD-8) up to 1994, and from 1995 onward, the 10th Revision (ICD-10) was utilized. A validation study showed that up to 83% of various primary diagnoses in the registry were correctly classified.(3) |
| Danish Medical Birth Register | 1973 | 1973 | Gestational age, parity, smoking*, body mass index** | Contains records of all births in Denmark with high completeness and validity for basic information such as maternal identification, parity, gestational age, birth weight, and Apgar score. Some data is sourced directly from the Civil Registration System.(4) |
| Statistics Denmark | 1966 | 1966 | Education level*** | Hosts social and demographic registries based on administrative data, considered highly reliable. |
| Danish National Prescription Registry | 1994 | 1995 | Hormonal  contraception,  ovarian stimulating drugs | Contains individual-level information on all prescription drugs dispensed at Danish pharmacies. Data entry is automated and barcode-based, ensuring high quality and completeness.(5) |
| ^*^ Height and weight information available from 2003. ^**^ Smoking information available from 1991.^***^ Education information available from 1981.  Abbreviations: ICD: International Classification of Diseases. | | | | |

# **REFERENCES**

1. Schmidt M, Pedersen L, Sørensen HT. The Danish Civil Registration System as a tool in epidemiology. Eur J Epidemiol. 2014;29(8):541-9.

2. Gjerstorff ML. The Danish Cancer Registry. Scand J Public Health. 2011;39(7_suppl):42-5.

3. Schmidt M, Schmidt SAJ, Sandegaard JL, Ehrenstein V, Pedersen L, Sørensen HT. The Danish National Patient Registry: a review of content, data quality, and research potential. Clin Epidemiol. 2015;7:449-90.

4. Bliddal M, Broe A, Pottegård A, Olsen J, Langhoff-Roos J. The Danish Medical Birth Register. Eur J Epidemiol. 2018;33(1):27-36.

5. Kildemoes HW, Sorensen HT, Hallas J. The Danish National Prescription Registry. Scand J Public Health. 2011;39(7 Suppl):38-41.
