# Supplementary material for: Leukemia in users of contemporary hormonal contraception: A nationwide registry-based cohort study among premenopausal women in Denmark
Source: PLoS Med. 2026 Jan 30;23(1):e1004652. doi: 10.1371/journal.pmed.1004652 (PMC12875577; doi:10.1371/journal.pmed.1004652)
Supplement: S2 Table — Abbreviations: ATC, Anatomical Therapeutic Chemical classification system; IUD, Intrauterine device. (DOCX) [file pmed.1004652.s002.docx]

| S2 Table. Exposure classification and ATC codes. | | | |
| --- | --- | --- | --- |
| Type | **Administration route** | **Form** | **ATC Codes** |
| Combined | **Oral** | Pills | G03AA01, G03AA03, G03AA05, G03AA07, G03AA09, G03AA10, G03AA11, G03AA12, G03AA14, G03AB03, G03AB04, G03AB05, G03AB06, G03AB08, G03HB01, G03AA16 |
| Combined | **Non-oral** | Vaginal ring, Patch | G02BB01, G03AA13 |
| Progestin-only | **Oral** | Pills | G03AC01, G03AC02, G03AC03, G03AC09, G033AC10 |
| Progestin-only | **Non-oral** | IUD, Injection, Implant | G02BA03, G03AC06, G03AC08 |
| Abbreviations: ATC: Anatomical Therapeutic Chemical classification system. IUD: Intrauterine device | | | |
