# Supplementary material for: Leukemia in users of contemporary hormonal contraception: A nationwide registry-based cohort study among premenopausal women in Denmark
Source: PLoS Med. 2026 Jan 30;23(1):e1004652. doi: 10.1371/journal.pmed.1004652 (PMC12875577; doi:10.1371/journal.pmed.1004652)
Supplement: S5 Table — *Adjusted for calendar year, age, and education. **Adjusted for calendar year, age, education, and body mass index in the first trimester of pregnancy. Abbreviations: CI, Confidence interval; IRR, Incidence rate ratio; PY, Person-years. Recent use: The six months following cessation of hormonal contraceptive use, as recorded in the prescription register. (DOCX) [file pmed.1004652.s005.docx]

| **S5 Table.** IRRs [95% CIs] for leukemia in Danish parous women aged 15–49 years, according to hormonal contraceptive use, adjusted for body mass index. | | | | |
| --- | --- | --- | --- | --- |
|  | **Any leukemia** | | | |
|  | **PY/100,000** | **Cases** | **IRR [95% CI]*** | **IRR [95% CI]**** |
| **Never use** | 12.7 | 12 | 1 [reference] | 1 [reference] |
| **Ever use** | 73.2 | 115 | 0.97 [0.51,1.83] | 0.96 [0.51,1.82] |
| **Current and recent use** | 48.3 | 62 | 0.96 [0.50,1.84] | 0.95 [0.49,1.83] |
| **Previous use** | 24.9 | 53 | 0.98 [0.50,1.94] | 0.98 [0.50,1.94] |
|  |  |  |  |  |
| *Adjusted for calendar year, age, and education | | | | |
| **Adjusted for calendar year, age, education, and body mass index in the first trimester of pregnancy. | | | | |
| Abbreviations: CI: Confidence interval. IRR: Incidence rate ratio. PY: Person-years. | | | | |
| Recent use: The six months following cessation of hormonal contraceptive use, as recorded in the prescription register. | | | | |
