# Supplementary material for: Leukemia in users of contemporary hormonal contraception: A nationwide registry-based cohort study among premenopausal women in Denmark
Source: PLoS Med. 2026 Jan 30;23(1):e1004652. doi: 10.1371/journal.pmed.1004652 (PMC12875577; doi:10.1371/journal.pmed.1004652)
Supplement: S6 Table — *Adjusted for calendar year, age, and education. **Adjusted for calendar year, age, education, and smoking in the first trimester of pregnancy. Abbreviations: CI, Confidence interval; IRR, Incidence rate ratio; PY, Person-years. Recent use: The six months following cessation of hormonal contraceptive use, as recorded in the prescription register. (DOCX) [file pmed.1004652.s006.docx]

| **S6 Table.** IRRs [95% CIs] for leukemia in Danish parous women aged 15–49 years, according to hormonal contraceptive use, adjusted for smoking. | | | | |
| --- | --- | --- | --- | --- |
|  | **Any leukemia** | | | |
|  | **PY/100,000** | **Cases** | **IRR [95% CI]*** | **IRR [95% CI]**** |
| **Never use** | 28.9 | 61 | 1 [reference] | 1 [reference] |
| **Ever use** | 102.3 | 211 | 1.09 [0.79,1.49] | 1.09 [0.79,1.50] |
| **Current and recent use** | 64.8 | 118 | 1.14 [0.81,1.61] | 1.15 [0.82,1.61] |
| **Previous use** | 37.5 | 93 | 1.02 [0.72,1.45] | 1.03 [0.72,1.46] |
|  |  |  |  |  |
| *Adjusted for calendar year, age, and education | | | | |
| **Adjusted for calendar year, age, education, and smoking in the first trimester of pregnancy. | | | | |
| Abbreviations: CI: Confidence interval. IRR: Incidence rate ratio. PY: Person-years. | | | | |
| Recent use: The six months following cessation of hormonal contraceptive use, as recorded in the prescription register. | | | | |
