# Supplementary material for: Leukemia in users of contemporary hormonal contraception: A nationwide registry-based cohort study among premenopausal women in Denmark
Source: PLoS Med. 2026 Jan 30;23(1):e1004652. doi: 10.1371/journal.pmed.1004652 (PMC12875577; doi:10.1371/journal.pmed.1004652)
Supplement: S8 Table — *Adjusted for calendar year, age, and education. Abbreviations: ALL, Acute lymphatic leukemia; AML, Acute myeloid leukemia; CLL, Chronic lymphatic leukemia; CML, Chronic myeloid leukemia; CI, Confidence interval; IRR, Incidence rate ratio; PY, Person-years. Recent use: The six months following cessation of hormonal contraceptive use, as recorded in the prescription register. Small cell suppression was applied in accordance with data protection guidelines from Statistics Denmark to prevent identification of individuals. (DOCX) [file pmed.1004652.s008.docx]

| **S8 Table.** IRRs [95% CIs] for types of leukemia in Danish women aged 15–49 years in 2000–2021, according to hormonal contraceptive use. | | | | | | | | | |
| --- | --- | --- | --- | --- | --- | --- | --- | --- | --- |
|  |  | **ALL** | | **AML** | | **CLL** | | **CML** | |
|  | **PY/100,000** | **Cases** | **IRR [95% CI]*** | **Cases** | **IRR [95% CI]*** | **Cases** | **IRR [95% CI]*** | **Cases** | **IRR [95% CI]*** |
| **Never use** | 48.8 | 26 | 1 [reference] | 56 | 1 [reference] | 30 | 1 [reference] | 26 | 1 [reference] |
| **Ever use** | 145.8 | 72 | 1.04 [0.64,1.69] | 151 | 1.05 [0.75,1.47] | 53 | 0.83 [0.50,1.83] | 74 | 1.02 [0.62,1.67] |
|  |  |  |  |  |  |  |  |  |  |
| **Current and recent use** | 90.2 | 47 | 1.02 [0.61,1.70] | 75 | 0.91 [0.63,1.33] | 28 | 1.05 [0.59,1.84] | 44 | 1.16 [0.68,1.98] |
| *Combined products* | 68.9 | 35 | 0.95 [0.55,1.63] | <60 | 0.94 [0.63,1.41] | 10 | 0.87 [0.41,1.83] | 30 | 1.22 [0.68,2.17] |
| Oral | 67.5 | 35 | 0.97 [0.56,1.66] | <60 | 0.95 [0.63,1.42] | 10 | 0.89 [0.42,1.86] | <30 | 1.17 [0.65,2.11] |
| Non-oral | 1.4 | 0 | . | <5 | 0.84 [0.11,6.16] | 0 | . | <5 | 3.89 [0.89,17.08] |
| *Progestin-only products* | 21.2 | 12 | 1.30 [0.62,2.73] | <20 | 0.84 [0.48,1.47] | 18 | 1.21 [0.63,2.34] | 14 | 1.05 [0.52,2.13] |
| Oral | 4.7 | <5 | 1.35 [0.40,4.59] | <5 | 0.91 [0.32,2.56] | <5 | 0.51 [0.07,3.82] | <5 | 1.31 [0.38,4.48] |
| Non-oral | 16.6 | <10 | 1.28 [0.57,2.90] | <20 | 0.82 [0.45,1.50] | <20 | 1.32 [0.68,2.57] | <15 | 1.00 [0.47,2.14] |
|  |  |  |  |  |  |  |  |  |  |
| **Previous use** | 55.6 | 25 | 1.09 [0.59,1.99] | 76 | 1.22 [0.84,1.77] | 25 | 0.67 [0.37,1.20] | 30 | 0.88 [0.50,1.55] |
| *Combined products* | 49.0 | <25 | 1.17 [0.63,2.17] | 65 | 1.18 [0.80,1.74] | <25 | 0.68 [0.37,1.25] | 25 | 0.88 [0.46,1.51] |
| *Progestin-only products* | 6.7 | <5 | 0.74 [0.17,3.21] | 11 | 1.45 [0.74,2.84] | <5 | 0.68 [0.23,2.02] | 5 | 1.10 [0.41,2.98] |
|  |  |  |  |  |  |  |  |  |  |
| *Adjusted for calendar year, age, and education. | | | | | | | | | |
| Abbreviations: ALL: Acute lymphatic leukemia. AML: Acute myeloid leukemia. CLL: Chronic lymphatic leukemia. CML: Chronic myeloid leukemia. CI: Confidence interval. IRR: Incidence rate ratio. PY: Person-years. | | | | | | | | | |
| Recent use: The six months following cessation of hormonal contraceptive use, as recorded in the prescription register. | | | | | | | | | |
| Small cell suppression was applied in accordance with data protection guidelines from Statistics Denmark to prevent identification of individuals. | | | | | | | | | |
