# Supplementary material for: Leukemia in users of contemporary hormonal contraception: A nationwide registry-based cohort study among premenopausal women in Denmark
Source: PLoS Med. 2026 Jan 30;23(1):e1004652. doi: 10.1371/journal.pmed.1004652 (PMC12875577; doi:10.1371/journal.pmed.1004652)
Supplement: S9 Table — *Adjusted for calendar year, age, and education. Abbreviations: CI, Confidence interval; IRR, Incidence rate ratio; PY, Person-years. Small cell suppression was applied in accordance with data protection guidelines from Statistics Denmark to prevent identification of individuals. (DOCX) [file pmed.1004652.s009.docx]

| **S9 Table.** Duration of recent and current use of oral contraceptives and the risk of acute myeloid leukemia. | | | |
| --- | --- | --- | --- |
|  | **Acute myeloid leukemia** | | |
|  | **PY/100,000** | **Cases** | **IRR [95% CI]*** |
| **Never use** | 78.6 | 91 | 1 [reference] |
|  |  |  |  |
| **Duration of current and recent use of oral contraceptives** |  |  |  |
| 0–5 years | 73.0 | 60 | 1.00 [0.70,1.43] |
| >5–10 years | 12.8 | <15 | 1.07 [0.58,1.97] |
| >10 years | 2.8 | <5 | 0.91 [0.28,2.93] |
|  | | | |
| *Adjusted for calendar year, age, and education | | | |
| Abbreviations: CI: Confidence interval. IRR: Incidence rate ratio. PY: Person-years. | | | |
| Small cell suppression was applied in accordance with data protection guidelines from Statistics Denmark to prevent identification of individuals. | | | |
