# Supplementary material for: Leukemia in users of contemporary hormonal contraception: A nationwide registry-based cohort study among premenopausal women in Denmark
Source: PLoS Med. 2026 Jan 30;23(1):e1004652. doi: 10.1371/journal.pmed.1004652 (PMC12875577; doi:10.1371/journal.pmed.1004652)
Supplement: S10 Table — *Adjusted for calendar year and education. Abbreviations: CI, Confidence interval; IRR, Incidence rate ratio; PY, Person-years. (DOCX) [file pmed.1004652.s010.docx]

| **S10 Table.** IRRs [95% CIs] for leukemia in Danish women, according to age group. | | | | |
| --- | --- | --- | --- | --- |
|  | **Any leukemia** | | |  |
|  | **PY/100,000** | **Cases** | **IRR [95% CI]*** | **P-value** |
| **Never use** | 78.6 | 241 | 1 [reference] |  |
|  |  |  |  |  |
| **Age (years)** |  |  |  |  |
| 15–20 | 40.3 | 65 | 0.82 [0.57,1.18] | 0.28 |
| 20–25 | 36.3 | 60 | 0.87 [0.61,1.24] | 0.44 |
| 25–30 | 30.9 | 59 | 1.01 [0.71,1.45] | 0.95 |
| 30–35 | 31.2 | 59 | 1 [reference] | 1 |
| 35–40 | 34.5 | 90 | 1.36 [0.98,1.89] | 0.07 |
| 40–45 | 36.1 | 147 | 2.12 [1.56,2.87] | <0.01 |
| 45–49 | 35.7 | 191 | 2.75 [2.05,3.71] | <0.01 |
|  | | | |  |
| *Adjusted for calendar year and education. | | | | |
| Abbreviations: CI: Confidence interval. IRR: Incidence rate ratio. PY: Person-years. | | | | |
