# Supplementary material for: Leukemia in users of contemporary hormonal contraception: A nationwide registry-based cohort study among premenopausal women in Denmark
Source: PLoS Med. 2026 Jan 30;23(1):e1004652. doi: 10.1371/journal.pmed.1004652 (PMC12875577; doi:10.1371/journal.pmed.1004652)
Supplement: S12 Table — * Using age as the underlying time scale in the Poisson regression. Abbreviations: ALL, Acute lymphatic leukemia; AML, Acute myeloid leukemia; CLL, Chronic lymphatic leukemia; CML, Chronic myeloid leukemia; CI, Confidence interval; IRR, Incidence rate ratio; PY, Person-years. Recent use: The six months following cessation of hormonal contraceptive use, as recorded in the prescription register. Small cell suppression was applied in accordance with data protection guidelines from Statistics Denmark to prevent identification of individuals. (DOCX) [file pmed.1004652.s012.docx]

| **S12 Table.** IRRs [95% CIs] for types of leukemia in Danish women aged 15–49 years, according to hormonal contraceptive use and unadjusted. | | | | | | | | | |
| --- | --- | --- | --- | --- | --- | --- | --- | --- | --- |
|  |  | **ALL** | | **AML** | | **CLL** | | **CML** | |
| **Hormonal contraceptive use** | **PY/100,000** | **Cases** | **IRR [95% CI]*** | **Cases** | **IRR [95% CI]*** | **Cases** | **IRR [95% CI]*** | **Cases** | **IRR [95% CI]*** |
| **Never use** | 78.6 | 39 | 1 [reference] | 91 | 1 [reference] | 47 | 1 [reference] | 42 | 1 [reference] |
|  |  |  |  |  |  |  |  |  |  |
| **Ever use** | 166.3 | 85 | 1.05 [0.71,1.57] | 175 | 0.99 [0.76,1.29] | 53 | 0.92 [0.62,1.37] | 77 | 0.99 [0.67,1.45] |
|  |  |  |  |  |  |  |  |  |  |
| **Current and recent use** | 106.9 | 56 | 0.99 [0.64,1.52] | 93 | 0.90 [0.66,1.22] | 28 | 1.10 [0.69,1.77] | 47 | 1.10 [0.71,1.71] |
| *Combined products* | 84.9 | 44 | 0.92 [0.58,1.46] | <75 | 0.97 [0.69,1.35] | 10 | 0.79 [0.40,1.58] | <35 | 1.08 [0.65,1.77] |
| Oral | 83.5 | 44 | 0.93 [0.59,1.47] | <75 | 0.97 [0.70,1.36] | 10 | 0.80 [0.40,1.60] | <35 | 1.03 [0.62,1.71] |
| Non-oral | 1.4 | 0 | . | <5 | 0.77 [0.11,5.53] | 0 | . | <5 | 3.97 [0.95,16.68] |
| *Progestin-only products* | 22.0 | <15 | 1.31 [0.67,2.54] | <25 | 0.73 [0.45,1.19] | <20 | 1.40 [0.81,2.41] | <20 | 1.14 [0.63,2.07] |
| Oral | 5.1 | <5 | 1.25 [0.38,4.08] | <5 | 0.72 [0.26,1.95] | <5 | 0.53 [0.07,3.86] | <5 | 1.62 [0.58,4.55] |
| Non-oral | 16.8 | <10 | 1.33 [0.63,2.78] | <20 | 0.73 [0.43,1.25] | <20 | 1.55 [0.89,2.70] | <15 | 1.03 [0.53,2.01] |
|  |  |  |  |  |  |  |  |  |  |
| **Previous use** | 59.4 | <30 | 1.20 [0.72,1.98] | 82 | 1.11 [0.82,1.51] | <30 | 0.78 [0.48,1.26] | 30 | 0.86 [0.54,1.39] |
| *Combined products* | 52.6 | <30 | 1.26 [0.75,2.12] | 71 | 1.10 [0.80,1.51] | <25 | 0.77 [0.46,1.29] | 25 | 0.82 [0.50,1.36] |
| *Progestin-only products* | 6.8 | <5 | 0.76 [0.18,3.18] | 11 | 1.21 [0.64,2.26] | <5 | 0.80 [0.29,2.21] | 5 | 1.13 [0.45,2.87] |
|  | | | | | | | | | |
| * Using age as the underlying time scale in the Poisson regression. | | | | | | | | | |
| Abbreviations: ALL: Acute lymphatic leukemia. AML: Acute myeloid leukemia. CLL: Chronic lymphatic leukemia. CML: Chronic myeloid leukemia. CI: Confidence interval. IRR: Incidence rate ratio. PY: Person-years. | | | | | | | | | |
| Recent use: The six months following cessation of hormonal contraceptive use, as recorded in the prescription register.  Small cell suppression was applied in accordance with data protection guidelines from Statistics Denmark to prevent identification of individuals. | | | | | | | | | |
