# Supplementary material for: Leukemia in users of contemporary hormonal contraception: A nationwide registry-based cohort study among premenopausal women in Denmark
Source: PLoS Med. 2026 Jan 30;23(1):e1004652. doi: 10.1371/journal.pmed.1004652 (PMC12875577; doi:10.1371/journal.pmed.1004652)
Supplement: S13 Table — * Using age as the underlying time scale in the Poisson regression. Abbreviations: CI, Confidence interval; IRR, Incidence rate ratio; PY, Person-years. Small cell suppression was applied in accordance with data protection guidelines from Statistics Denmark to prevent identification of individuals. (DOCX) [file pmed.1004652.s013.docx]

| **S13** **Table.** Use of hormonal contraception by duration of use and time since last use and the risk of leukemia and unadjusted. | | | |
| --- | --- | --- | --- |
|  | **Any leukemia** | | |
| **Hormonal contraceptive use** | **PY/100,000** | **Cases** | **IRR [95% CI]*** |
| **Never use** | 78.6 | 241 | 1 [reference] |
| **Duration of use** |  |  |  |
| **Any hormonal contraception** |  |  |  |
| 0–5 years | 88.0 | 185 | 0.95 [0.77,1.16] |
| >5–10 years | 15.6 | 50 | 1.21 [0.88,1.65] |
| >10 years | 3.2 | 8 | 0.71 [0.35,1.43] |
| ***Combined*** |  |  |  |
| 0–5 years | 69.7 | 125 | 0.88 [0.70,1.11] |
| >5–10 years | 12.5 | <40 | 1.32 [0.93,1.87] |
| >10 years | 2.7 | <10 | 0.67 [0.30,1.51] |
| ***Progestin-only*** |  |  |  |
| 0–5 years | 18.3 | 60 | 1.10 [0.83,1.47] |
| >5–10 years | 3.1 | <15 | 0.93 [0.51,1.70] |
| >10 years | 0.5 | <5 | 0.85 [0.21,3.42] |
|  |  |  |  |
| **Time since last use** |  |  |  |
| **Any hormonal contraception** |  |  |  |
| 0–5 years | 36.6 | 102 | 1.04 [0.82,1.32] |
| >5–10 years | 14.0 | 51 | 1.10 [0.81,1.49] |
| >10 years | 8.9 | 34 | 0.92 [0.64,1.32] |
|  | | | |
| * Using age as the underlying time scale in the Poisson regression. | | | |
| Abbreviations: CI: Confidence interval. IRR: Incidence rate ratio. PY: Person-years.  Small cell suppression was applied in accordance with data protection guidelines from Statistics Denmark to prevent identification of individuals. | | | |
